# 5-azacytidine reduces methylation, promotes differentiation and induces tumor regression in a patient-derived IDH1 mutant glioma xenograft

**DOI:** 10.18632/oncotarget.1408

**Published:** 2013-09-16

**Authors:** Alexandra Borodovsky, Vafi Salmasi, Sevin Turcan, Armida W. M. Fabius, Gilson S. Baia, Charles G. Eberhart, Jon D. Weingart, Gary L. Gallia, Stephen B. Baylin, Timothy A. Chan, Gregory J. Riggins

**Affiliations:** ^1^ Department of Neurosurgery, Johns Hopkins University School of Medicine, Baltimore, MD, USA; ^2^ Human Oncology and Pathogenesis Program, Memorial Sloan-Kettering Cancer Center, New York, New York, USA; ^3^ Department of Pathology, Johns Hopkins University School of Medicine, Baltimore, MD, USA; ^4^ Department of Oncology, Johns Hopkins University School of Medicine, Baltimore, MD, USA

**Keywords:** IDH, 5-azacytidine, progressive glioma, xenograft, astrocytoma, methylation

## Abstract

Somatic mutations in *Isocitrate Dehydrogenase 1* (*IDH1*) are frequent in low grade and progressive gliomas and are characterized by the production of 2-hydroxyglutarate (2-HG) from α-ketoglutarate by the mutant enzyme. 2-HG is an “oncometabolite” that competitively inhibits α-KG dependent dioxygenases resulting in various widespread cellular changes including abnormal hypermethylation of genomic DNA and suppression of cellular differentiation. Despite the growing understanding of *IDH* mutant gliomas, the development of effective therapies has proved challenging in part due to the scarcity of endogenous mutant *in vivo* models. Here we report the generation of an endogenous *IDH1* anaplastic astrocytoma model which rapidly grows *in vivo*, produces 2-HG and exhibits DNA hypermethylation. Using this model, we have demonstrated the preclinical efficacy and mechanism of action of the FDA approved demethylating drug 5-azacytidine *in vivo*. Long term administration of 5-azacytidine resulted in reduction of DNA methylation of promoter loci, induction of glial differentiation, reduction of cell proliferation and a significant reduction in tumor growth. Tumor regression was observed at 14 weeks and subsequently showed no signs of re-growth at 7 weeks despite discontinuation of therapy. These results have implications for clinical trials of demethylating agents for patients with *IDH* mutated gliomas.

## INTRODUCTION

Gliomas are a classification of nervous system tumors arising from glial cells, the most common of which are astrocytomas and oligodendrogliomas, arising from astrocytes and oligodendroglia, respectively. The most aggressive glioma is the WHO grade IV astrocytoma, or glioblastoma multiforme (GBM), which can arise *de novo* (primary GBM) or can progress from a WHO grade II or III glioma (secondary or progressive GBM).

Whole exome sequencing of GBMs revealed frequent mutations at the position 132 arginine residue in the catalytic domain of *Isocitrate Dehydrogenase 1* (*IDH1*) of progressive gliomas and targeted sequencing found additional mutations in the mitochondrial family member *IDH2* [1, 2]. Remarkably, more than 70% of grade II-III gliomas and secondary GBMs were found to bear mutations in *IDH1* or *IDH2*. Both the *IDH1* and *IDH2* mutations were highly conserved, confined to a single residue, R132 and R172, respectively, and most frequently a single allele was mutated with the wild type allele retained [1]. Since its identification as an oncogene in glioma, *IDH* mutations have been identified in a number of cancer types, including a large percentage of acute myeloid leukemia and myelodysplastic syndromes [3, 4] as well as a small percentage of prostate cancer [5], cholangiocarcinoma [6, 7], and chondrosarcoma [8]. Overall evidence suggests that gain-of-function *IDH1* or *IDH2* mutations affect one or several fundamental cellular processes that underlie several types of malignancies.

Subsequent mechanistic studies revealed the probable mechanism of this oncogene: IDH mutations alter the enzymatic function of the protein to produce D-2-hydroxyglutarate (2-HG) from α-ketoglutarate (α-KG) [9]. Normally not present in human cells at significant concentrations, 2-HG functions as an “oncometabolite” that competitively inhibits several α-KG dependent dioxygenases [10]. Of particular importance, 2-HG has been shown to inhibit both the TET family of 5-methylcytosine hydroxylases and the H3K9 demethylase KDM4C, leading to the accumulation of repressive histone and DNA methylation alterations and a subsequent transcriptional block of differentiation gene expression [11, 12]. It has recently been shown that IDH mutations alone are sufficient to induce a global hypermethylated phenotype that is characteristic of the gliomas with these mutations [12, 13].

Abnormal DNA hypermethylation has been recognized as a possible target in cancer and DNA methylation reducing drugs, including 5-azacytidine that was reported nearly 40 years ago [14]. 5-azacytidine is an analogue of cytidine and it is incorporated into DNA and RNA. At therapeutic doses, 5-azacytidine inhibits DNA methyltransferase leading to a reduction in DNA methylation. In particular, 5-azacytidine is a potent inhibitor of DNA methyltransferase 1 (DNMT1), inducing ubiquitin-dependent degradation of the protein [15].

Unfortunately, despite the growing understanding of *IDH* mutant gliomas, the development of effective therapies has proved challenging. *IDH* mutant tumors do not adapt well to growth in culture, including ‘stem cell’ media and endogenous mutant models remain scarce [16, 17]. Engineered cell lines have been useful in elucidating the complex network underlying *IDH* mutations but lack the appropriate genetic and mutational context found in patient derived *IDH* mutant gliomas. Patient derived endogenous *IDH* mutant models are therefore critical for the development and testing of therapies which target *IDH* driven oncogenenic pathways and mechanisms.

Here we report the generation of an endogenous *IDH1* anaplastic astrocytoma and the preclinical demonstration of efficacy and mechanism of 5-azacytidine in this model. Long term administration of 5-azacytidine resulted in inhibition of DNMT1, loss of methylation of key genomic markers, *in vivo* induction of differentiation, reduction of cell proliferation and a significantly reduced tumor growth. Tumor growth was essentially arrested at 14 weeks and subsequently showed no signs yet of re-growth after removal of the therapy.

## RESULTS

### Establishment and serial passage of an IDH1 (R132H) anaplastic astrocytoma model

**Figure 1:**
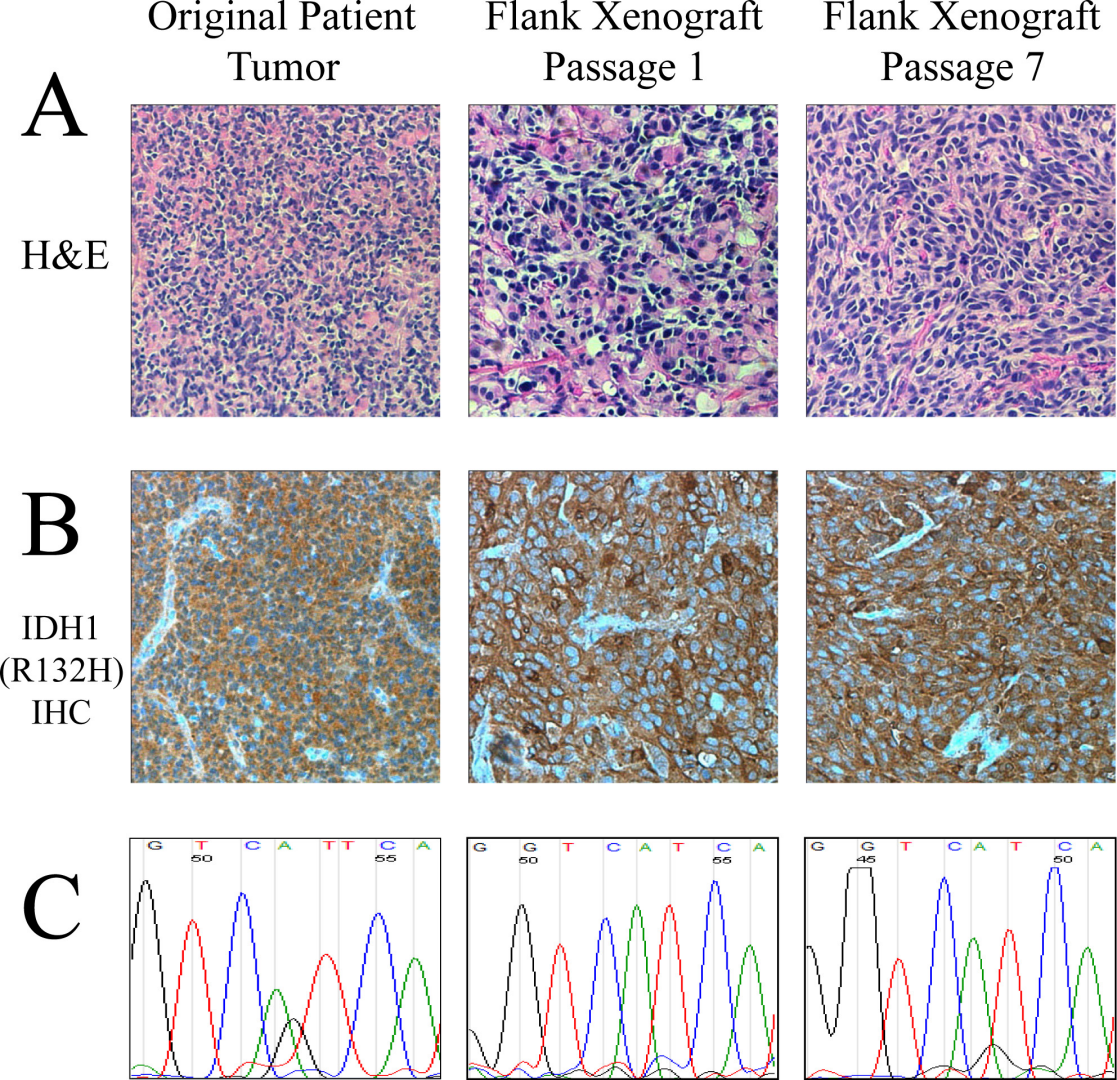
Characteristic histological and genetic features of the IDH1 (R132H) anaplastic astrocytoma model (A) H&E sections of the primary tumor and subsequent flank xenografts show histopathological similarity to the original tumor including a typical astrocytic morphology, gemistocytic cells and mitotic figures. (B) Immunohistochemical staining specific for the IDH1 (R132H) mutant protein shows robust staining in the original tumor and all subsequent xenografts. (C) Sequencing of exon 4 of IDH1 shows an initial heterozygous G/A mutation in the original patient tumor which converts to a hemizygous genotype when the wild type copy is lost in the xenograft.

Tumor was obtained from the resection of an anaplastic astrocytoma (WHO grade III) from an adult patient with a history of glioma who presented with a large right temporal lobe tumor. The patient had been previously diagnosed with a low grade astrocytoma (WHO grade II) after his initial craniotomy twelve years prior. The resected WHO grade III tumor was found to have a high degree of anaplasia but lacked areas of necrosis and vascular proliferation. Direct sequencing of *IDH1* exon 4 demonstrated that the grade III tumor bore a heterozygous G395A (R132H) mutation (Figure 1C), validated by immunohistochemistry (Figure 1B). Fresh tissue was collected from the specimen and directly implanted subcutaneously into athymic nude mice. Additionally, neurosphere cultures from the same patient were attempted in multiple media conditions including serum-free media containing hFGF and hEGF, but these cells did not propagate. However, a first generation tumor quickly arose in the nude mouse approximately one month after implantation as a large, localized subcutaneous mass. The resulting IDH1 (R132H) tumor model was designated as JHH-273. Direct sequencing of the xenograft tissue revealed preservation of the *IDH1* (G395A) mutation, but revealed a loss of the wild type copy (Figure 1C). Immunohistochemistry demonstrated strong IDH1 (R132H) expression throughout the tissue (Figure 1B). All subsequent serial passages have retained the hemizygous IDH1 (R132H) mutation and exhibit robust expression of the mutant protein.

### Xenografts show histopathological similarity and diffuse growth pattern

Histopathological analysis of H&E samples obtained from the patient's recurrent tumor sample and subsequently xenograft tissue showed that both the flank and orthotopic xenografts maintain histopathological similarity to the patient tumor and maintain morphological characteristics of anaplastic astrocytomas (Figure 1A). The patient's recurrent tumor was a cellular infiltrating astrocytoma with scattered gemistocytic cells as well as rare tumor giant cells. Scattered mitotic figures were identified consistent with the diagnosis of an anaplastic astrocytoma. Immunostaining for mutant IDH1 was strongly and diffusely positive. JHH-273 xenografts maintained many of the features of the primary tumor including a typical astrocytic morphology as well as scattered tumor giant cells, mixed with vascular, stromal, and skeletal muscle elements characteristic of this anatomical site.

Intracranial injections showed infiltrative growth throughout the cortex and deep grey matter structures (data not shown).

### JHH-273 grows *in vivo* but not *in vitro*

**Figure 2:**
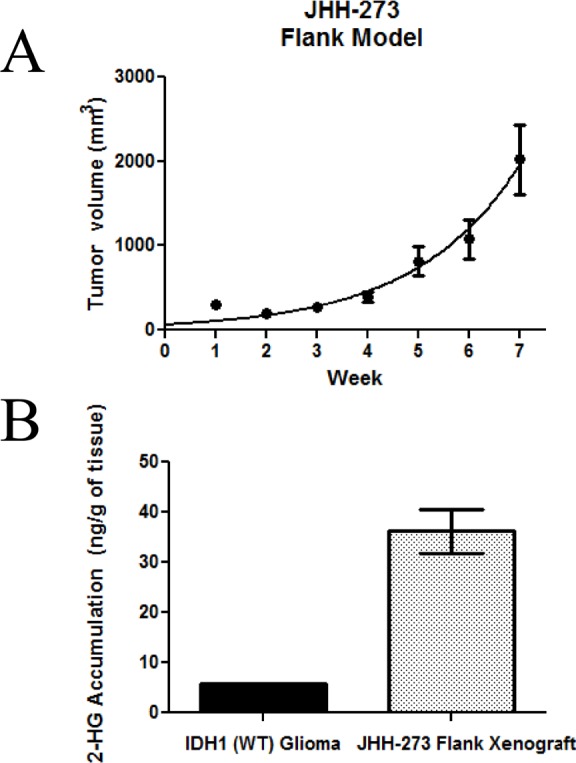
Growth *in vivo* and 2-HG production in the IDH mutant model (A) Subcutaneously implanted flank xenografts grow to maximum size (2.0 cm^3^) in approximately 7 weeks. (B) *IDH* mutant xenograft produces high levels of 2-HG as measured by LC/MS. Error bars=SEM.

The JHH-273 model grows rapidly in the flank (Figure 2A). Serially transplantable flank xenografts reach maximum size (2.0 cm^3^) approximately 7 weeks after implantation and grow as dense, localized, hypercellular masses.

Production of 2-HG from α-KG is a hallmark of *IDH* mutations and its accumulation is believed to underlie the pathogenesis of *IDH* mutations. Therefore, we investigated whether our patient-derived model produces 2-HG. LC/MS analysis was performed on snap frozen tissue from well-established flank xenograft (passage 7). JHH-273 produced high levels of 2-HG which correspond to the endogenous levels reported in *IDH1/2* mutant gliomas (Figure 2B). In contrast, 2-HG was nearly undetectable in tissue obtained from *IDH1* wild type glioma controls.

### JHH-273 CpG hypermethylation and by 5-azacytidine treatment *in vivo*

**Figure 3:**
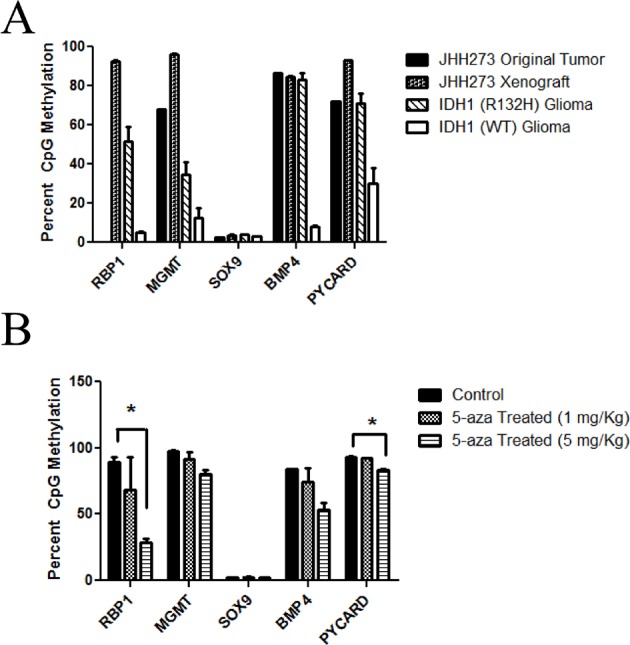
JHH-273 shows characteristic DNA hypermethylation which can be reversed with 5-azacytidine treatment *in vivo* (A) Pyrosequencing shows that the original patient tumor exhibits high levels of DNA methylation in several target genes. This hypermethylated phenotype is maintained in the xenograft and is characteristic of *IDH1* mutant gliomas. In contrast, IDH1 wild type glioma is not hypermethylated. (B) 5-azacytidine treatment for 1 cycle reverses methylation at several targets in a dose specific manner. * p<0.05, Error bars=SEM.

Recent data has shown that one consequence of *IDH* mutation is the induction of global DNA hypermethylation which is believed to contribute to tumor formation and maintenance [12, 21, 22]. In order to determine the methylation status of JHH-273, we performed pyrosequencing analysis of bisulfate-converted genomic DNA at five genetic loci. Targets were selected based on the reported frequency of hypermethylation in IDH1 (R132H) primary tissue and engineered cell systems as well as their known functional impact in tumoriogenesis or tumor maintenance [21-31]. The analysis was performed using genomic DNA obtained from both early and late passage xenograft tissue (passages 1, 7 and 10) as well as two unrelated IDH1 (R132H) anaplastic astrocytoma samples and three IDH1 (WT) glioma primary patient samples as controls. The original patient tumor exhibited high degrees of CpG methylation at three of the five loci (the RBP1 locus did not amplify), which was maintained in the xenograft tissue at all passages analyzed (Figure 3A). The high levels of CpG methylation were consistent with other primary patient IDH1 (R132H) anaplastic astrocytoma samples. In contrast, wild type *IDH1* gliomas exhibited low levels of methylation at all targets analyzed, consistent with reported data. Of note, although *SOX9* hypermethylation in *IDH* mutant gliomas has been reported in several studies, we did not detect significant methylation in either primary tissue sample or xenograft. Overall, JHH-273 reflects the hypermethylated genomic landscape of the original patient tumor and characteristic of an IDH1 (R132H) anaplastic astrocytoma.

To determine whether hypomethylating agent 5-azacytidine is able to reverse methylation of the JHH-273 tumors *in vivo*, we analyzed CpG methylation of flank tumors following treatment with one cycle of 5-azacytidine (1 mg/kg and 5 mg/kg). 5-azacytidine was able to reduce CpG hypermethylation at four of the five targets analyzed in a dose specific manner (Figure 3B). Although a significant tumor regression was observed in the high dose treatment group, this was accompanied with significant toxicity (data not shown).

### Long term treatment with 5-azacytidine reduces tumor growth in an IDH1 mutant model

**Figure 4:**
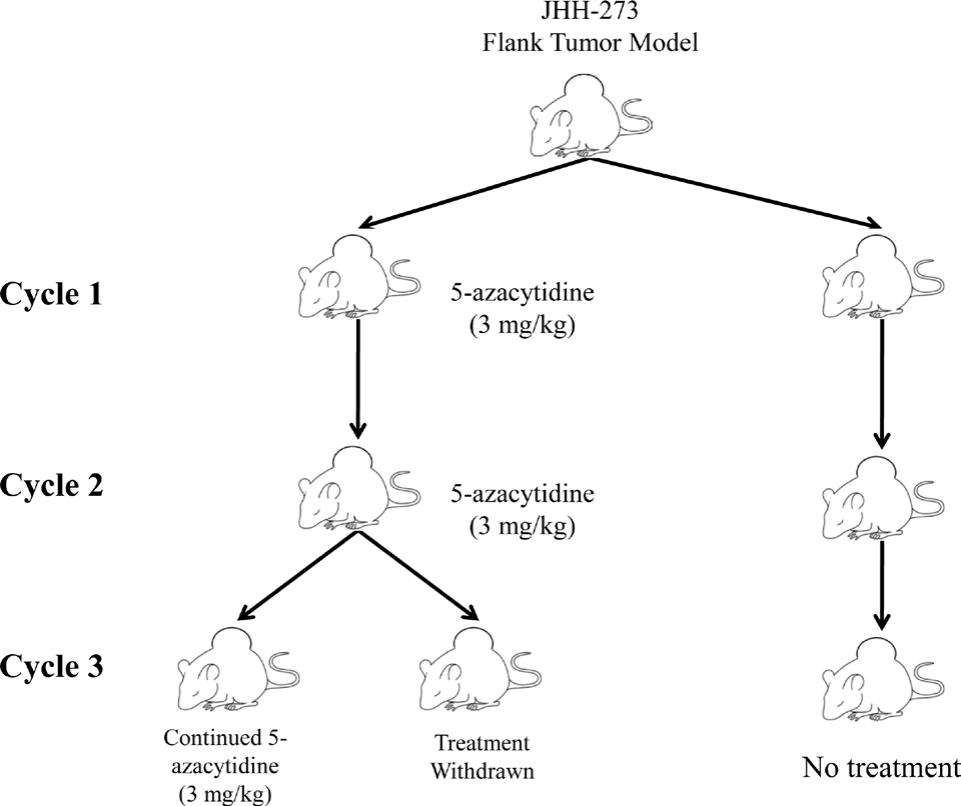
Treatment strategy for 5-azacytidine in the IDH1 mutant flank model Flank tumors were treated with 5-azacytidine starting 5 days after implantation until maximum tumor size was reached (Cycle 1). In order to extend the relative time of treatment, the flank tumors were individually passaged and 5-azacytidine treatment resumed immediately following implantation (Cycle 2). Tumor passaging was repeated a third time into two groups, one which immediately resumed 5-azacytidine treatment or one which had treatment withdrawn (Cycle 3).

**Figure 5:**
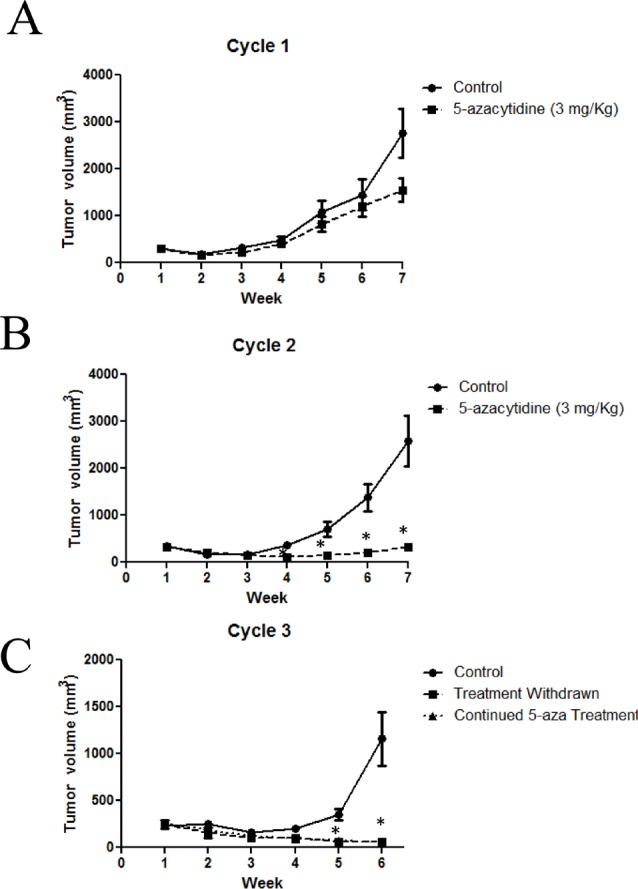
Long term treatment with 5-azacytidine reduces tumor growth in an IDH1 mutant model (A) Mice bearing IDH1 mutant flank tumors and treated with 5-azacytidine show a reduction in tumor burden (B) Cycle 2 of 5-azacytidine treatment significantly reduces tumor growth compared to untreated tumors (C) Pre-treatment with 5-azacytidine arrests tumor growth, even after treatment is withdrawn. Cycles 1 and 2: Five mice (10 tumors) per treatment group, Cycle 3: Four mice (4 tumors) per treatment group. * p<0.01, Error bars=SEM,

To extend the relative exposure time of JHH-273 tumor to 5-azacytidine, serial passage of flank tumors from treated mice was performed using the strategy outlined in Figure 4. A moderate reduction of tumor burden was observed within one treatment cycle, but this decrease was not significant (p=0.27) (Figure 5A). Following passage of 5-azacytidine treated flank tumors, a second cycle of treatment with the 5-azacytidine was resumed as before and continued until maximum tumor burden was reached. 5-azacytidine significantly reduced tumor burden by the fourth week of Cycle 2 (p<0.01), and the difference between untreated and treated increased throughout the treatment period (Figure 5B). During passage of tumor after Cycle 2, the tissue was found to be unusually firm and encapsulated by gelatinous tissue. Enzymatically disassociated fractions of the tumor were found to be less than 10% viable by Trypan blue exclusion (data not shown).

5-azacytidine serially treated tumors were implanted for a third time into two groups of athymic nude mice (Cycle 3). To assess the durability of the therapeutic response, treatment was withdrawn in one group. Significant tumor regression was observed in both groups, including the treatment withdrawn group, suggesting a durable therapeutic response of 5-azacytidine (Figure 5C).

### Treatment with 5-azacytidine induces differentiation and reduces the proliferative index

**Figure 6:**
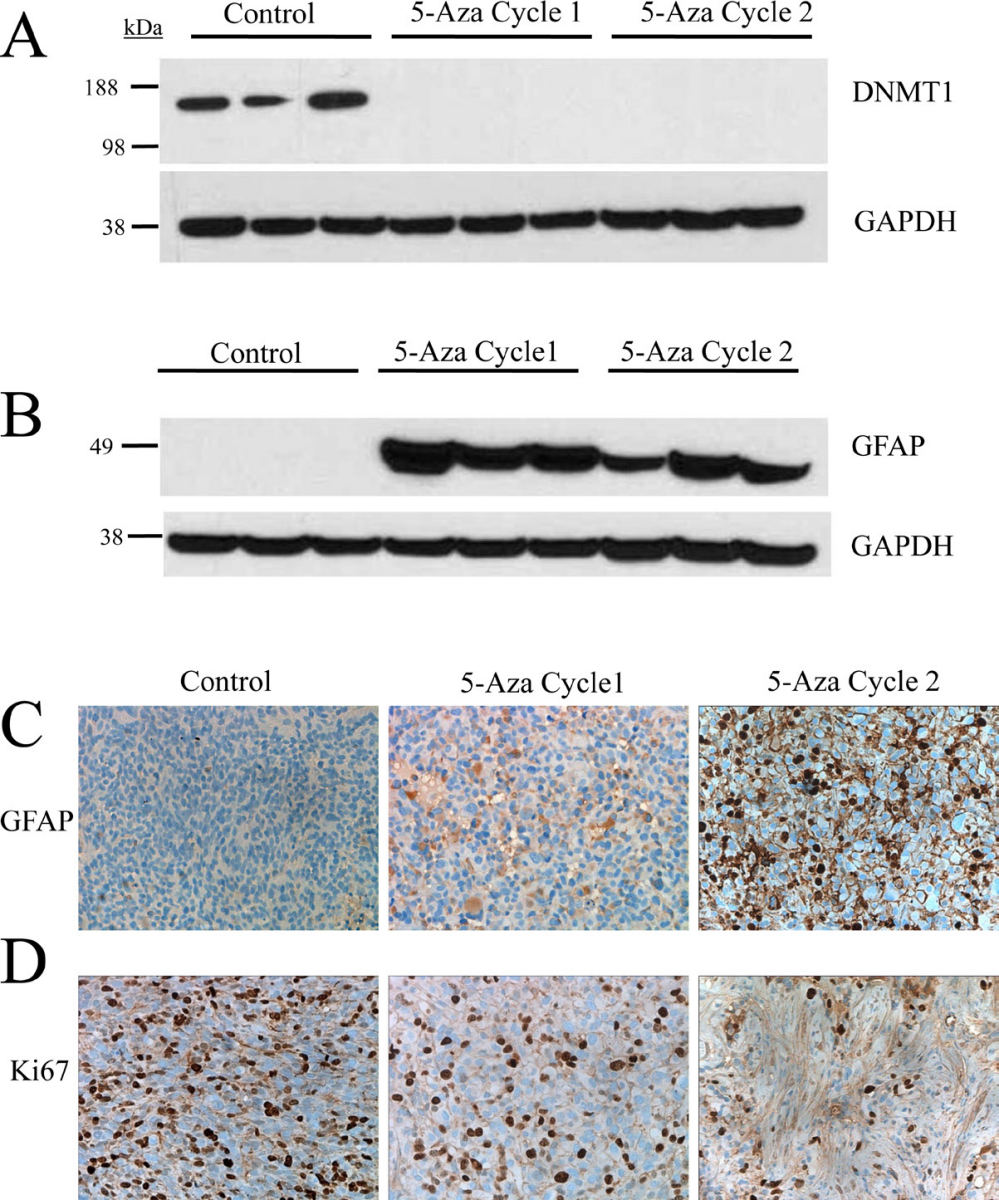
Treatment with 5-azacytidine induces differentiation and reduces the proliferative index in an *in vivo* IDH1 (R132H) glioma model (A) 5-azacytidine treatment causes loss of DNMT1 expression *in vivo* following one treatment cycle. (B) GFAP expression is restored following one passage of 5-azacytidine treatment and is maintained. (C) Immunohistological staining of GFAP shows significant increase of protein expression in the cytoplasm of 5-azacytidine treated cells (D) Ki67 staining shows a decrease in the proliferative index of 5-azacytidine treated cells in a time dependent manner.

Intracellular 5-azacytidine activity was confirmed by DNMT1 expression in tissue from untreated and 5-azacytidine treated tumors (Cycles 1 and 2). DNMT1 is expressed at high levels in untreated tumors but expression of the protein is robustly inhibited following treatment with 5-azacytidine. DNMT1 protein was undetectable after one treatment cycle of 5-azacytidine (Figure 6A).

Prior studies had proposed that the *IDH* mutation inhibits DNA and histone demethylation leading to a block in cellular differentiation [24]. Additionally in a study published in this issue, treatment of endogenous *IDH1* mutant glioma cell lines with hypomethylating agent 5-aza-2'-deoxycytidine was found to drive differentiation *in vitro* [32]. To test whether reduction of hypermethylation by 5-azacytidine leads to an increase in cellular differentiation *in vivo*, immunoblotting was performed for glial fibrillary acidic protein (GFAP) in tumors treated with 5-azacytidine. GFAP expression was undetectable in the untreated tumors, confirming the undifferentiated phenotype of IDH (R132H) tumors. Following one cycle of 5-azacytidine treatment, GFAP expression was robustly increased and protein levels were maintained throughout the second treatment cycle. To validate these findings, immunohistochemical analysis was performed on formalin fixed paraffin embedded tissue. Compared with untreated tissue, 5-azacytidine treatment markedly increased the fraction of GFAP-positive cells (Figure 6C). These results confirm that IDH mutations plays an active role in repressing cellular differentiation, a deficiency which is reversible with long term treatment with hypomethylating agent 5-azacytidine.

To test whether 5-azacytidine induced differentiation translated to a decrease in proliferation, tumors from 5-azacytidine treated mice were stained with the proliferation marker Ki-67. 5-azacytidine treatment significantly reduced the fraction of Ki-67 positive cells in a time dependent manner, with the greatest decrease in proliferation during the 7 to 14 week period of Cycle 2 (Figure 6D).

## DISCUSSION

Somatic mutations in *IDH* are found in a high percentage of low grade and progressive gliomas. *IDH* mutant gliomas are associated with a pro-neural gene expression profile, a characteristic pattern of DNA hypermethylation and a signature of repressive histone methylation [12]. A mechanism by which the 2-HG produced by IDH mutations are believed to promote tumorigenesis is by blocking cellular differentiation via hypermethylation of tumor suppressor genes involved in differentiation [11-13, 21, 22].

The current therapy for low grade gliomas is surgical resection followed by monitoring with periodic MRI scans. Unfortunately, the majority of these tumors recur or progress to a high grade glioma. Although repeat surgery and additional treatment with radiation can temporarily slow tumor growth, if the gliomas progress to high grade no curative therapy is available. Median survival for grade II astrocytoma (fibrillary or diffuse astrocytoma) is approximately 5 to 7 years and for grade III astrocytoma (anaplastic), median survival is 4 to 5 years [33].

In this work, we report the first *in vivo* model of a patient derived *IDH* mutant anaplastic astrocytoma, JHH-273. This model was established after many attempts, which underscores the difficulty most labs have had at growing *IDH* mutant astrocytomas. The goal of establishing the xenografts was to enhance translational studies with more accurate models harboring relevant mutations that developed during the course of human tumorigenesis. Importantly, we show in that IDH1 (R132H) expression is stable through multiple passages, 2-HG is robustly produced in the tissue, and the model bears a hypermethylated CpG phenotype characteristic of *IDH1* mutant glioma.

Once evidence emerged that hypermethylation was a likely oncogenic mechanism of *IDH* mutations, demethylating agents became an attractive choice for translational investigation. When 5-azacytidine treatment was first tested in the JHH-273 model, a decrease in methylation was observed in a dose specific manner but was associated with only marginal reduction of tumor burden. We were able to achieve a significant *in vivo* response by lowering drug dosing and extending the relative exposure time of tumor to drug by passaging pre-treated tumor tissue into treatment naïve mice.

Short term 5-azacytidine treatment (1 cycle, 7 weeks) perhaps started to slow tumor growth, but by doubling treatment time, we were able to achieve tumor regression. Treatment with 5-azacytidine for 2 cycles elicited a durable treatment response as treatment withdrawal for an additional 6 weeks did not produce any visible signs of tumor re-growth (although we continue to monitor the mice). At the end of 2 treatment cycles, the tumors had changed dramatically in appearance with the presence of hard fibrous tissue and less than 10% cell viability. Additionally, with 7 weeks of treatment 5-azacytidine drives cellular differentiation to the astrocytic linage as seen by increase in GFAP expression, further underscoring the reversal of the presumed mechanism of mutant IDH1 oncogenesis. These data are consistent with the hypothesis that demethylating drugs may promote re-expression of previously silenced Polycomb controlled genes and subsequently activate genes involved in differentiation [21, 34].

The observed induction of cellular differentiation and subsequent reduction in proliferation following treatment with a hypomethylating agent in *IDH* mutant glioma is surprisingly consistent with an independent study that is simultaneously reported in this journal [32]. In this companion work, decitabine, a closely related demethylating agent, was found to preferentially induce differentiation in *IDH* mutant glioma cells but not in wild type cells. This work showed very similar mechanistic results to this study, and since both the drug and the models utilized are independent, the collective evidence supports the hypothesis that the gene expression reactivated by demethylating agents is sufficient to produce terminal differentiation in the self renewing malignant cells of the tumor.

5-azacytidine is currently FDA approved for the treatment of myelodysplastic syndrome and well tolerated in patients. Its close structural analogue, decitabine, can effectively cross the blood brain barrier in laboratory animals [35]. Experimentally, pre-treatment of cells with transient, low dose exposure of 5-azacytidine has been shown to decrease tumorigenicity and percentage of stem-like cells in several cancer models [36]. In addition to considering 5-azacytidine or decitabine for recurrent or high grade *IDH* mutant glioma, 5-azacytidine therapy may also be useful as a maintenance therapy following tumor resection. Due to the infiltrative growth pattern of astrocytoma, complete resection is nearly impossible and the remaining tumor frequently recurs, often as a higher grade glioma. Low-dose treatment with 5-azacytidine following resection may drive the remaining mutant *IDH* glioma cells into differentiation, thereby delaying recurrence.

This work supports the further investigation of demethylating drugs such as 5-azacytidine for its use against mutant *IDH* gliomas both in the laboratory and in clinical trials. Although it is very likely that these drugs have the potential to help patients with IDH1 mutant gliomas, the optimal demethylating drug, patient population, dosing strategy, delivery and drug combinations have yet to be determined.

## METHODS

### Flank xenograft establishment and passage

A fresh tissue sample was obtained during the resection of an anaplastic astrocytoma (WHO grade III) from a male patient. The tissue was implanted subcutaneously into a 4–6-week old female athymic nude mice (NCI-Frederick) under a Johns Hopkins approved protocol as previously described [18]. Briefly, the freshly harvested tumor tissue was mechanically dissociated using scalpels, mixed with an equal volume of growth factor–reduced Matrigel (BD Biosciences, CA), and injected subcutaneously into the flanks of athymic nude mice (0.2cc/flank). Xenografts were passaged in a similar fashion and animals were monitored frequently for signs of tumor growth. All animal protocols and procedures were performed in accordance with the Johns Hopkins Animal Care and Use Committee guidelines. Orthotopic tumors were established using cells obtained from flank tissue disassociated using a 2:1 ratio of collagenase (10mg/mL, Invitrogen, NY) and hyaluronidase (1000 units/mL, Sigma Aldrich, MO). Cross-sectional samples were obtained at each passage and fixed in formalin. The samples were then embedded in paraffin and stained by H&E or used for immunohistochemistry. The IDH1 (R132H) mutation was validated by direct sequencing for each passage.

### Sequencing of IDH1

Genomic DNA was isolated from patient tissue and flank xenografts using the DNeasy Blood and Tissue Kit (Qiagen, CA) according to manufacturer's instructions. PCR and sequencing was conducted as previously described [19]. Briefly, 60 ng of genomic DNA was added to a standard PCR reaction to amplify a portion of exon 4 of *IDH1* (forward 5'-GTAAAACGACGGCCAGTTGAGCTCTATATGCCATCACTGC 3', reverse 5'-CAATTTCATACCTTGCTTAATGGG-3'). The PCR product was purified using the QIAquick Gel Extraction Kit (Qiagen, CA) and submitted for sequencing (Genewiz, NJ) using targeted primers (forward 5'-CGGTCTTCAGAGAAGCCATT-3', and reverse 5'-GCAAAATCACATTATTGCCAAC-3').

### LC/MS

2-hydroxyglutarate levels were analyzed from snap frozen flank xenograft samples. Prior to extraction, frozen samples were thawed in a water bath at ambient temperature. Tissue homogenates were prepared at a concentration of 200 mg/mL in methanol. A 10 μL aliquot of homogenized tissue was added to a borosilicate glass test tube (13×100 mm) containing 10 μL of acetonitrile solution and 2-phosphonomethyl pentanedioic acid (1 mg/mL), which was used as the internal standard. The tube was mixed vigorously and evaporated under nitrogen gas at 4°C until completely dried. After the samples were dried, 100 μl of acetonitrile and 100 μL of N-tert-Butyldimethysilyl-N-methyltrifluoro-acetamide were added sequentially. The tubes were incubated at 80°C for 1 hour then diluted 1:10 with acetonitrile. 100 μl of the top layer was transferred to a 250-μL polypropylene autosampler vial sealed with a Teflon crimp cap. 10 μL of each sample was injected onto the LC/MS/MS for quantitative analysis using a temperature-controlled autosampling device operating at 10°C. Chromatographic analysis was performed using an ACQuity ™ UItra Performance LC (Waters, MA). Separation of the analyte from potentially interfering material was achieved at ambient temperature using X-Terra^®^ RP18 column (20 × 2.1 mm, Waters, MA) packed with a 3.5 μm RP_18_ material (Milford, MA). The mobile phase used for the chromatographic separation was composed of acetonitrile with 0.1% formic acid and 2mM ammonium acetate in water (80:20, v/v) and delivered using an isocratic flow rate of 0.3 mL/minute. The column effluent was monitored using a QTRAP ^R^ 5500 mass spectrometer (AB SCIEX, MA). The instrument was equipped with an electrospray interface, operated in a positive mode and controlled by the Analyst 1.5.1 software. The mass spectrometry was programmed to monitor the following MRM's 491.0 to 359 for 2HG and 683.0 to 551.4 for the IS. Samples were quantified over the assay range of 0.02 to 2 μg/mL. The standard curve of ratio response (analyte peak area/IS peak area) vs. concentration was plotted using linear regression with 1/x weighting for the data analyzed.

### Histology and Immunohistochemistry

Paraffin-embedded (5 micron) sections were deparaffinized, and stained with either hematoxylin and eosin (H&E) or immunohistochemical stains as specified. Heat-induced epitope retrieval was performed for 36 minutes at 98°C in target retrieval buffer. Immunohistochemical staining was performed using antibodies specific for IDH1 (R132H) (Dianova, DIAH09LM), GFAP (Dako, Z0334) and Ki-67 (Ventana, 790-4286). Immunostaining was visualized using the ultraView DAB detection system (Ventana Medical Systems, AZ).

### Pyrosequencing of target genes

Bisulfite conversion was carried out using EpiTect Bisulfite Kit (Qiagen, CA) with 1μg genomic DNA. PCR was carried out using PyroMark PCR kit (Qiagen, CA) following the manufacturer's instructions. The samples were run in a 25 μL reaction with 2.5 μL of primer reconstituted according to manufacturer's instructions. The thermocycler conditions are an initial activation step of 95°C for 15 minutes, 45 cycles of 94°C for 30 seconds, 56°C for 30 seconds, and 72°C for 30 seconds, and a final extension of 72°C for 10 minutes. Controls included 100%, 50%, and 0% human methylated control DNA (Zymo Research Corp, CA), and a no-template control. Samples plus controls were amplified using primer kits Hs_PYCARD_03_PM, Hs_RBP1_02_PM, Hs_MGMT_01_PM, Hs_SOX9_08_PM, and Hs_BMP4_02_PM (Qiagen, CA). Amplified DNA was then pyrosequenced using the PyroMark CpG Assay kit (Qiagen, CA) on the PyroMark 24 system (Qiagen, CA). Bisulfite conversion, PCR amplification and pyrosequencing was performed at the Genetic Resources Core Facility, Johns Hopkins Institute of Genetic Medicine, Baltimore, MD.

### Therapeutic administration of 5-azacytidine to JHH-273 flank bearing mice

Female, athymic nude mice aged 4-6 weeks were implanted with the JHH-273 tumor line as described above. Five days after implantation, mice received daily *i.p.* injections of 5-azacytidine (Sigma Aldrich, MO; 3 mg/kg) diluted in sterile water for five days followed by a two day rest period. Treatment continued until tumors reached maximum allowable size (Cycle 1). Following sacrifice, tumors were harvested and passaged individually into another set of female athymic nude mice, identically as before. 5-azacytidine treatment was resumed immediately after tumor implantation and continued until tumors reached maximum allowable size (Cycle 2). Following the second cycle of treatment, tumors were again resected and passaged into two groups of mice. To assess the durability of the therapeutic response, 5-azacytidine treatment was withdrawn in one group of pre-treated animals and resumed immediately in the other (Cycle 3). Treatment strategy is outlined in Figure 4. Tumors were measured weekly and volume was calculated as: H×L×W (mm^3^).

### Immunoblotting

Protein lysates were prepared using RIPA buffer and immunoblot analysis was performed as previously described [20]. Briefly, protein lysates were obtained from freshly resected flank tumors using RIPA buffer (ThermoScientific, MA) containing Halt protease inhibitor cocktails (Pierce, IL) according to the recommendations of the manufacturer. Lysates (50 μg) were heated to 95°C in Laemmli sample buffer for 10 min and separated on SDS polyacrylamide gels. Proteins were transferred to polyvinylidene fluoride membranes (Bio-Rad) in Western transfer buffer [25 mmol/L Tris (pH 8.3), 192 mmol/L glycine, and 20% methanol]. For Western blot analysis, membranes were blocked for 1 h at room temperature in 5% nonfat dry milk in TBST (1× TBS, 0.1% Tween 20) and incubated overnight at 4°C with antibodies against GFAP (Cell Signaling Technology, MA), or DNMT1 (New England Biolabs, MA). After washing, membranes were incubated with a horseradish peroxidase-linked goat anti-rabbit antibody for 1 h at room temperature. Antibody detection was achieved by chemiluminescence according to the manufacturer's recommendations (Pierce, IL).

### Statistical analysis

Statistical analyses were performed using a Student t-test for comparisons between the treatment groups. A p value of < 0.05 was considered significant.
